# Genomic determinants and an exploratory prognostic model for immunotherapy outcomes in recurrent or metastatic cervical cancer

**DOI:** 10.1093/oncolo/oyag236

**Published:** 2026-06-22

**Authors:** Lingling Gu, Biqing Zhu, Cuicui Liu, Xiaoying Wu, Yaru Zhang, Jiani Yin, Fufeng Wang, Tingting Hu, Yaqin Wu

**Affiliations:** Department of Medical Image Center, Jiangsu Cancer Hospital, Jiangsu Institute of Cancer Research, The Affiliated Cancer Hospital of Nanjing Medical University, Nanjing 210000, China; Department of Radiation Oncology, Jiangsu Cancer Hospital, Jiangsu Institute of Cancer Research, The Affiliated Cancer Hospital of Nanjing Medical University, Nanjing 210000, China; Geneseeq Research Institute, Nanjing Geneseeq Technology Inc, Nanjing 210032, China; Geneseeq Research Institute, Nanjing Geneseeq Technology Inc, Nanjing 210032, China; Geneseeq Research Institute, Nanjing Geneseeq Technology Inc, Nanjing 210032, China; Geneseeq Research Institute, Nanjing Geneseeq Technology Inc, Nanjing 210032, China; Geneseeq Research Institute, Nanjing Geneseeq Technology Inc, Nanjing 210032, China; Department of Nursing, Jiangsu Cancer Hospital, Jiangsu Institute of Cancer Research, The Affiliated Cancer Hospital of Nanjing Medical University, Nanjing 210000, China; Department of Radiation Oncology, Jiangsu Cancer Hospital, Jiangsu Institute of Cancer Research, The Affiliated Cancer Hospital of Nanjing Medical University, Nanjing 210000, China

**Keywords:** cervical cancer, immunotherapy, biomarkers, genomics, progression-free survival

## Abstract

**Background:**

Immune checkpoint inhibitors (ICIs) improve outcomes in recurrent or metastatic cervical cancer, but responses are heterogeneous and biomarkers beyond programmed death-ligand 1 (PD-L1) are limited.

**Materials and Methods:**

Targeted sequencing of 437 cancer-related genes was performed in 42 patients receiving ICI-based therapy. Genomic correlates of progression-free survival (PFS) were evaluated, and an exploratory prognostic model was developed using least absolute shrinkage and selection operator regression and multivariable Cox regression. The Cancer Genome Atlas (TCGA) cohort was used for transcriptomic analysis.

**Results:**

The cohort included 42 patients, with a median age of 51 years; 85.7% were human papillomavirus-positive, 90.5% had squamous cell carcinoma, and 54.8% had a programmed death-ligand 1 combined positive score (PD-L1 CPS) ≥5. Frequent alterations included *PIK3CA* (57.1%), *FBXW7* (21.4%), *TERT* (21.4%), *BAP1* (19.0%), and *EP300* (16.7%). Tumor mutational burden-associated mutations in *PIK3CA*, *EP300*, *CREBBP*, and *TERT* were associated with prolonged PFS (*P* < .001), whereas PD-L1–associated mutations showed numerically longer PFS (*P* = .170). *EP300* (*P* = .007), *PIK3CA* (*P* < .01), and homologous recombination repair pathway alterations (*P* = .016) were associated with favorable PFS, whereas *KEAP1* (*P* < .01) and *TP53* (*P* = .010) mutations were associated with shorter PFS. A five-feature genomic model stratified patients into high- and low-risk groups (*P* < .001, 1-year AUC = .889). In the exploratory cohort, high-risk patients showed numerically shorter PFS (*P* = .060). In TCGA samples, high-risk tumors showed enrichment of angiogenesis, epithelial-mesenchymal transition, hypoxia, inflammatory response, and tumor necrosis factor-α/nuclear factor-κB signaling.

**Conclusion:**

Specific genomic alterations may help stratify immunotherapy outcomes in recurrent or metastatic cervical cancer. The proposed genomic risk model remains exploratory and requires validation in larger, independent cervical cancer cohorts.

Implications for Practice Genomic profiling may complement PD-L1 testing to refine risk stratification in recurrent or metastatic cervical cancer receiving immunotherapy. *PIK3CA*, *EP300*, and homologous recombination pathway alterations were associated with favorable PFS, whereas *KEAP1* and *TP53* alterations were associated with shorter PFS. High-risk genomic features may help identify patients who require closer monitoring, while exploratory transcriptomic findings provide biological context for future validation studies.

## Introduction

Cervical cancer remains one of the leading causes of cancer-related morbidity and mortality among women globally, despite advances in screening and human papillomavirus (HPV) vaccination.^1^^,^^2^ As HPV vaccination initiatives continue to expand, particularly with the transition from quadrivalent to nonavalent vaccines, the epidemiological landscape of cervical cancer may continue to evolve. This transition is bringing increased clinical attention to tumors associated with less common HPV subtypes, non-squamous histologies, and HPV-negative disease.^3–5^ Because these subgroups are frequently characterized by distinct clinical and molecular features that often confer a poorer prognosis, there is an urgent need for improved molecular characterization and precise therapeutic stratification in recurrent or metastatic cervical cancer. Historically, platinum-based chemotherapy with or without bevacizumab provided only modest survival benefits for patients with recurrent or metastatic cervical cancer.^6^ More recently, immune checkpoint inhibitors (ICIs) have reshaped first-line and subsequent-line management: pembrolizumab combined with chemotherapy (± bevacizumab) improved progression-free and overall survival (OS) in KEYNOTE-826, establishing PD-1–based therapy as a new standard for PD-L1–positive tumors.^7^ In the post-platinum setting, PD-(L)1 inhibitors (e.g. pembrolizumab in PD-L1–positive disease and cemiplimab irrespective of PD-L1 status) have demonstrated clinically meaningful benefit.^8^^,^^9^ Nevertheless, responses are heterogeneous and durable benefit remains limited to a subset of patients, underscoring the need for predictive biomarkers beyond PD-L1. PD-L1 expression quantified by combined positive score (CPS) is the most commonly used enrichment marker in cervical cancer, yet it is imperfect: PD-L1–positive tumors can be non-responders, while a fraction of PD-L1–negative tumors respond.^7–9^ Tumor mutational burden (TMB) and microsatellite instability (MSI) are established biomarkers across cancers, but MSI-high is rare in cervical cancer, and the predictive value of intermediate TMB is uncertain.^10^^,^^11^ Cervical carcinogenesis is strongly HPV-driven and is characterized by the expression of viral xenoantigens, such as HPV E6 and E7 oncoproteins, which may serve as immunogenic targets and contribute to antitumor immune responses even in the absence of markedly elevated TMB. HPV-associated cervical cancer is also characterized by prominent APOBEC-mediated mutagenesis, which may further increase neoantigen load and shape the tumor immune microenvironment.^12–14^ In addition, recurrent alterations in pathways such as PI3K, NOTCH, RTK–RAS, TP53, and homologous recombination (HR pathway) have been described, yet their relationships to ICI outcomes in cervical cancer are not well defined.^12^^,^^15^ Taken together, there is a compelling rationale to integrate genomic features with immunologic readouts to refine patient selection for ICIs.

Here, we comprehensively characterized the mutational landscape of recurrent or metastatic cervical cancer treated with ICIs. Based on these findings, we developed a prognostic model to stratify patients by risk and further evaluated the immunologic relevance of key genomic alterations in an independent The Cancer Genome Atlas (TCGA) cohort. This integrative approach not only offers mechanistic insight into how specific genomic changes may influence ICI responsiveness but also provides a potential framework for patient stratification in cervical cancer immunotherapy.

## Materials and methods

### Patient cohort and clinical data collection

This retrospective study included 42 patients with histologically confirmed recurrent or metastatic cervical cancer who received immunotherapy at Jiangsu Cancer Hospital between January 2019 and July 2023. All patients were treatment-naïve at the time of tumor tissue collection. Clinical data, including age, histological subtype, HPV status, treatment history, and PD-L1 expression, were collected. PD-L1 expression was assessed using the Dako PD-L1 IHC 22C3 pharmDx kit and CPS, with CPS ≥1 considered positive. This retrospective study was approved by the Ethics Committee of Jiangsu Cancer Hospital (No. BK20220735) and conducted in accordance with the Declaration of Helsinki. The requirement for written informed consent was waived due to the study’s retrospective design.

### Sample collection, sequencing, and mutation calling

Formalin-fixed, paraffin-embedded tumor tissues and matched peripheral blood samples were collected at baseline. DNA extraction and sequencing library preparation were performed as previously described.^16^ The GeneseeqPrime^TM^ panel (Nanjing Geneseeq Technologies Inc.) was used for targeted sequencing of 437 cancer-related genes (Table S1). Sequencing was performed on Illumina HiSeq4000 platform. Low-quality reads were removed using Trimmomatic (v0.39), and the remaining reads were aligned to the hg19 reference genome using Burrows-Wheeler Aligner (v0.7.12). Deduplication, local realignment, and base quality recalibration were performed according to Genome Analysis Toolkit best practices. Somatic mutations were called using VarScan2, and variants with a variant allele frequency ≥2% and at least five supporting reads were retained. Known sequencing artifacts were removed using an internally generated artifact list and matched normal controls.

TMB was calculated as the number of nonsynonymous mutations per megabase, and high TMB was defined as TMB ≥10 muts/Mb according to the KEYNOTE-158 study.^17^ The chromosomal instability score (CIS) was defined as the proportion of DNA segments with a log2 ratio > ±0.2 in all the covered genomic regions.^18^ Co-occurrence or mutual exclusivity between gene pairs was evaluated using Fisher’s exact test with false discovery rate (FDR) adjustment. Alterations in genes involved in the cGAS/STING pathway genes covered by the sequencing panel (*IFNA6* and *IFNB1*) were evaluated according to the KEGG MEDICUS reference cGAS/STING signaling pathway gene set.

### Immunotherapy efficacy assessment

Disease progression was determined based on radiological and clinical assessments during routine follow-up. Progression-free survival (PFS) was defined as the interval from immunotherapy initiation to the disease progression or death from any cause. OS was defined as the interval from immunotherapy initiation to death from any cause, with patients alive at last follow-up censored at last contact. Associations between gene mutations, pathway alterations, mutational signatures, and PFS were evaluated using univariable Cox proportional hazards regression.

### Construction and evaluation of the genomic prognostic model

Univariable Cox regression was first used to evaluate clinical and genomic features. Variables significant in univariable analysis were subjected to least absolute shrinkage and selection operator (LASSO) regression to reduce collinearity and select candidate predictors. The optimal penalty parameter λ was selected using 5-fold cross-validation, and variables with non-zero coefficients at lambda.min were incorporated into the multivariable Cox model. A prognostic risk score was constructed as a linear combination of the retained variables weighted by Cox regression coefficients.

Model performance was evaluated in terms of discrimination, calibration, and clinical utility. Discrimination was assessed using the concordance index (C-index) and time-dependent area under the receiver operating characteristic (ROC) curve. The C-index was used to evaluate the overall discriminative ability of the model, whereas time-dependent ROC curves and corresponding AUCs were used to assess predictive accuracy for 1-year and 2-year PFS. Calibration plots were generated to compare predicted and observed survival probabilities. Decision curve analysis (DCA) was performed to evaluate the clinical net benefit of the model across different threshold probabilities. Time-dependent ROC curves and AUCs were calculated using the timeROC package. The C-index was obtained using functions from the survival package. Calibration plots were generated using the rms package. DCA was performed using the rmda package. To further assess model stability and internal consistency, internal validation was performed using 1000-fold bootstrap resampling and leave-one-out cross-validation (LOOCV). All analyses were performed using R software version 4.2.0. Patients were stratified into high- and low-risk groups based on the median risk score. The model was exploratorily assessed in a publicly available pan-cancer cohort of 249 ICI-treated patients, of whom 245 had available PFS data.^19^ The same cutoff was applied, and survival differences were evaluated using Kaplan-Meier analysis and log-rank tests.

### Transcriptomic and tumor microenvironment analysis

RNA sequencing data from 286 cervical cancer samples in the TCGA cohort were analyzed. Differential expression analysis was performed using the R package DESeq2. Gene set enrichment analysis (GSEA) was conducted using clusterProfiler with Hallmark gene sets, and pathways with FDR-adjusted *P-*value < .050 were considered significant.^20^ Immune cell infiltration was computationally estimated using the CIBERSORT^21^ based on TCGA bulk transcriptomic data, tumor microenvironment scores using xCell^22^ from TIMER2.0 (http://timer.cistrome.org/), and T-cell dysfunction and exclusion scores using Tumor Immune Dysfunction and Exclusion (TIDE, http://tide.dfci.harvard.edu).

### Statistical analysis

Continuous variables were summarized as medians with ranges, and categorical variables as counts and percentages. Between-group differences were assessed using the Wilcoxon rank-sum test or Fisher’s exact test, as appropriate. Kaplan-Meier survival curves were generated, and hazard ratios with 95% confidence intervals (CI) were estimated using Cox proportional hazards models. For variables with sparse events or complete/quasi-complete separation in standard Cox regression, Firth’s penalized Cox regression was applied. A two-sided *P-*value < .05 was considered statistically significant. Multiple testing correction was applied where appropriate using the FDR method. Analyses were performed using R software (version 4.2.0).

## Results

### Baseline characteristics and molecular features of the study cohort

A total of 42 patients with recurrent or metastatic cervical cancer receiving immunotherapy were included. Baseline clinical characteristics were summarized in Table 1. The median age was 51 years (range, 30-73), with 57.1% of patients aged over 50 years. Patients (85.7%) were HPV-positive, and 90.5% had squamous cell carcinoma. The median PD-L1 CPS was 5 (range, 1-92). Among the included patients, 26 patients (61.9%) received chemotherapy plus immunotherapy, and 16 patients (38.1%) received chemotherapy plus immunotherapy plus bevacizumab. Regarding the specific ICIs, 24 patients (57.1%) received pembrolizumab and 18 patients (42.9%) received cadonilimab. None of the baseline clinical characteristics, including age, HPV status, histological subtype, PD-L1 CPS cutoffs, and treatment regimen, was significantly associated with PFS (all *P* > .05; Table S2).

**Table 1:** Baseline characteristics and treatment regimens of the study cohort.

Characteristics	Cervical cancer (*n* = 42)
**Age at diagnosis**	
**≤50**	18 (42.9%)
**>50**	24 (57.1%)
**Median (range, years)**	51 (30-73)
**HPV status**	
**Negative**	6 (14.3%)
**Positive**	36 (85.7%)
**Histological subtype**	
**Adenocarcinoma**	4 (9.5%)
**Squamous cell carcinoma**	38 (90.5%)
**PD-L1 (CPS)**	
**<5**	19 (45.2%)
**≥5**	23 (54.8%)
**Median(range)**	5 (1-92)
**Treatment regimen**	
**Chemotherapy + Immunotherapy**	26 (61.9%)
**Chemotherapy + Immunotherapy + Bevacizumab**	16 (38.1%)
**Immune checkpoint inhibitors**	
**Pembrolizumab**	24 (57.1%)
**Cadonilimab**	18 (42.9%)

Abbreviations: HPV, human papillomavirus; CPS, combined positive score; PD-L1, programmed death-ligand 1.

**Figure 1:**
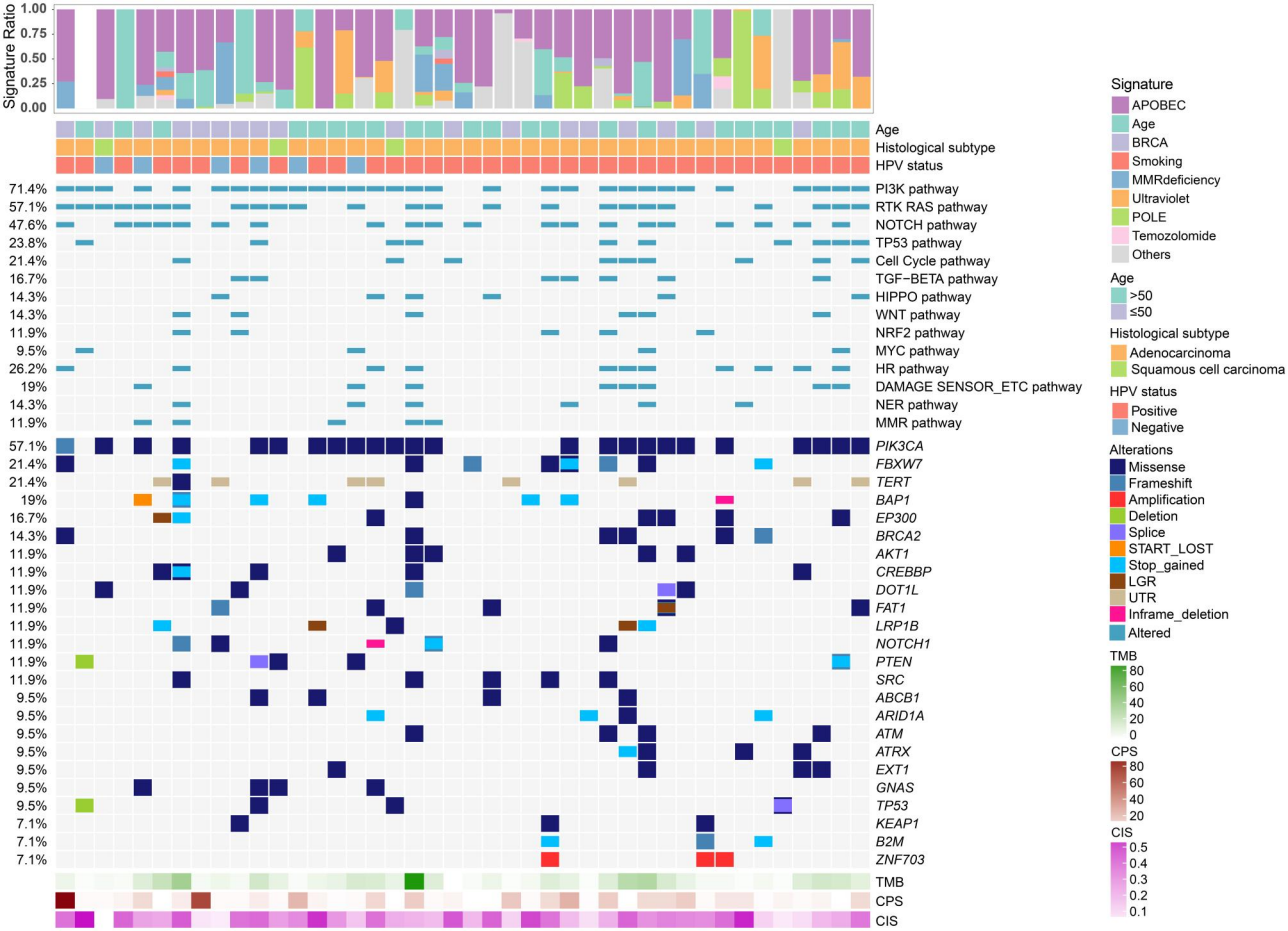
Integrated molecular and clinical landscape of the cohort. Each column represents an individual patient. The upper bar plot shows the distribution of mutational signatures, followed by panels depicting clinical characteristics, including age, histological subtype, and human papillomavirus status, alterations in major signaling pathways, and gene-level mutations. The bottom heatmaps illustrate tumor mutational burden, programmed death-ligand 1 combined positive score, and chromosomal instability score. HPV, human papillomavirus; TMB, tumor mutational burden; PD-L1, programmed death-ligand 1; CPS, combined positive score; CIS, chromosomal instability score; CSCC, cervical squamous cell carcinoma; CADC, cervical adenocarcinoma; MMR, mismatch repair.

All patients had at least one detectable mutation, with a median of 8 mutations per patient (range, 2-54). To reduce the influence of rare events, subsequent analyses focused on recurrent mutations detected in at least three patients. The most frequently mutated genes were *PIK3CA* (57.1%), *FBXW7* (21.4%), *TERT* (21.4%), *BAP1* (19.0%), and *EP300* (16.7%) (Figure 1). Co-mutation analysis identified significant co-occurrence of *SRC* with *NFE2L2*, *CREBBP* with *FOXL2*, and *PRDM1* with *ATM* (all FDR-adjusted *P* < .05; Figure S1). At the pathway level, alterations most frequently involved PI3K (71.4%), RTK_RAS (57.1%), NOTCH (47.6%), and TP53 (23.8%) signaling pathways. APOBEC activity was the predominant mutational signature (73.8%). The median TMB was 9.3 muts/Mb (range, 0-87.5), and the median CIS was 0.318 (range, 0.018-0.58; Figure 1).

**Figure 2:**
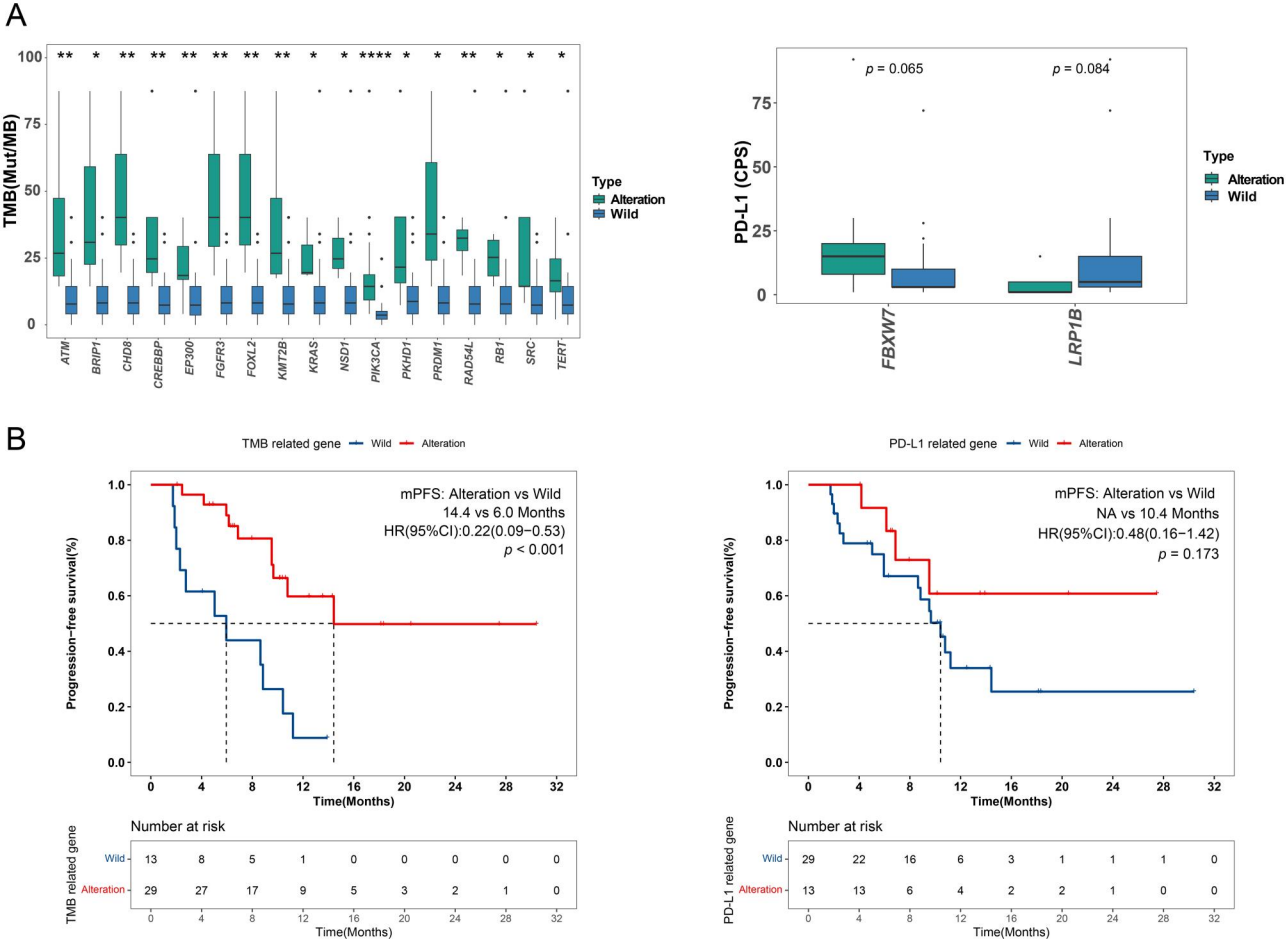
Associations between mutation-defined biomarker groups, tumor mutational burden, programmed death-ligand 1 expression, and progression-free survival. (A) Boxplots showing associations between specific gene alterations and continuous tumor mutational burden or programmed death-ligand 1 combined positive score values. (B) Kaplan-Meier curves comparing progression-free survival between patients with and without tumor mutational burden-associated mutations or programmed death-ligand 1-associated mutations. TMB, tumor mutational burden; PD-L1, programmed death-ligand 1; CPS, combined positive score; PFS, progression-free survival; mPFS, median progression-free survival; HR, hazard ratio; CI, confidence interval; NA, not available.

Given that TMB and PD-L1 CPS are established biomarkers related to immunotherapy response, we further explored the associations between specific gene mutations and the continuous values of these two biomarkers. Significantly higher TMB values were observed in patients harboring mutations in *CREBBP* (median TMB, 24.7 vs 7.4 muts/Mb, *P* = .002), *EP300* (18.5 vs 7.4 muts/Mb, *P* = .006), *PIK3CA* (14.4 vs 3.65 muts/Mb, *P* < .001), *SRC* (14.4 vs 7.4 muts/Mb, *P* = .040), and *TERT* (16.5 vs 7.4 muts/Mb, *P* = .040), compared with their respective wild-type tumors (Figure 2A). *FBXW7*-mutant tumors showed numerically higher PD-L1 CPS values, whereas *LRP1B*-mutant tumors showed numerically lower PD-L1 CPS values, although neither association was statistically significant (*P* = .065 and *P* = .084, respectively; Figure 2A). Based on our observations and previous studies,^23–31^  *PIK3CA*, *CREBBP*, *SRC*, *TERT*, and *EP300* mutations were classified as TMB-associated mutations, whereas *FBXW7* and *LRP1B* mutations were classified as PD-L1–associated mutations for subsequent analyses in this study.

Given the known interaction between HPV E6/E7 and p53,^32^^,^^33^ we further assessed *TP53* mutation by HPV status. *TP53* mutations were detected in 4 patients, with a numerically higher frequency in HPV-negative tumors than in HPV-positive tumors (16.7% [1/6] vs 8.3% [3/36]), although this difference was not significant (*P* = .474).

### Efficacy of immunotherapy and related biomarkers

The median follow-up duration was 13.5 months (range, 1.7-30.4). At data cutoff, 21 patients (50.0%) had experienced disease progression, whereas OS data remained immature, with deaths recorded in 3 patients (7.1%). The median PFS was 10.4 months (95% CI: 8.8–not reached); given the limited number of OS events, subsequent prognostic modeling was based on PFS. Patients with TMB-associated mutations had significantly longer PFS than wild-type tumors (mPFS: 14.42 vs 5.95 months, HR = 0.22, 95% CI: 0.09-0.53, *P* < .001), whereas PD-L1-associated mutations showed numerically longer but non-significant PFS (mPFS: not reached vs 10.41 months, HR = 0.48, 95% CI: 0.16-1.42, *P* = .170; Figure 2B).

**Figure 3:**
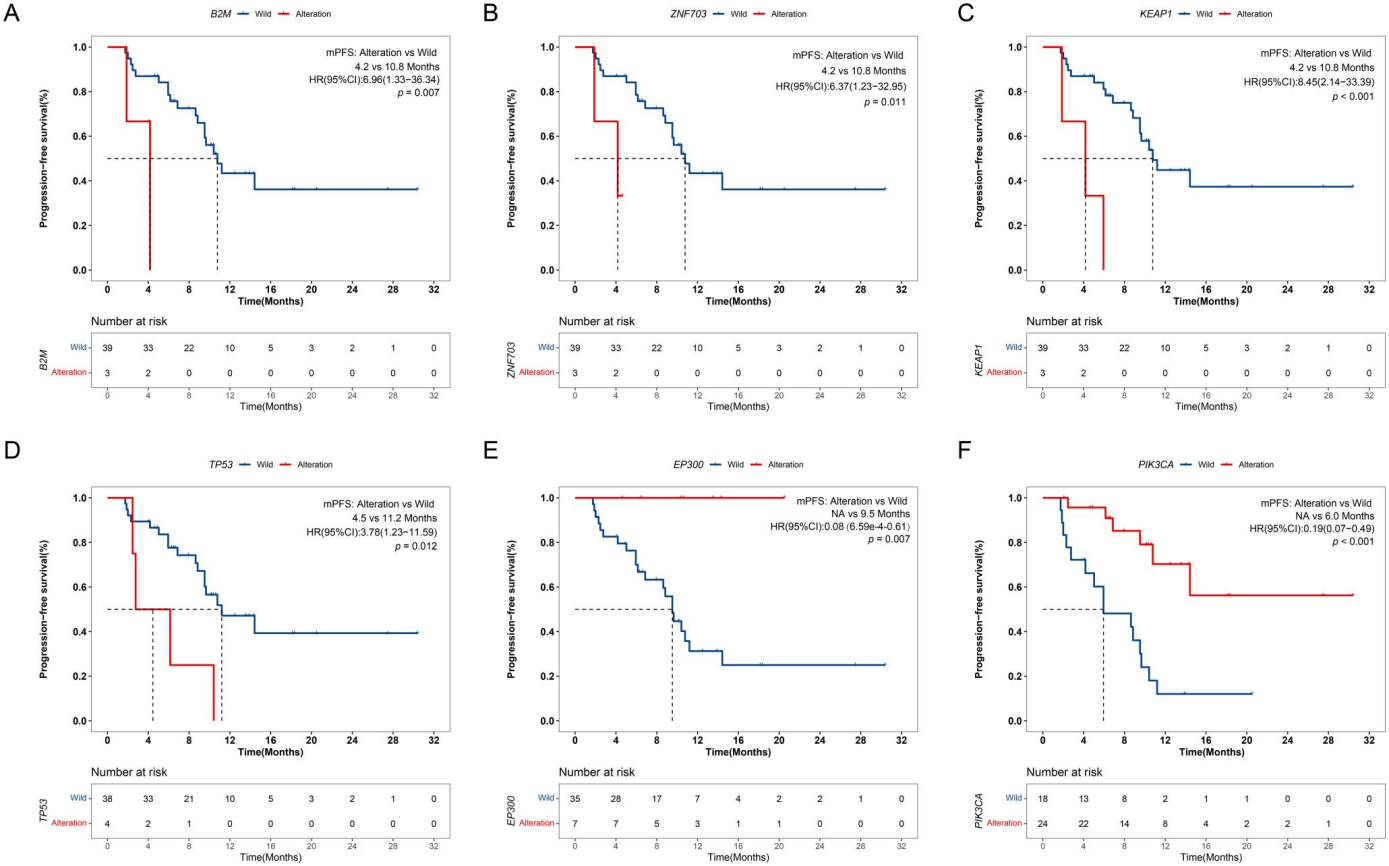
Progression-free survival according to individual genomic alterations. Kaplan-Meier curves showing progression-free survival according to alteration status of (A) *B2M*, (B) *ZNF703*, (C) *KEAP1*, (D) *TP53*, (E) *EP300*, and (F) *PIK3CA*. PFS, progression-free survival; mPFS, median progression-free survival; HR, hazard ratio; CI, confidence interval; NA, not available.

Univariable Cox regression was performed to evaluate associations between genomic features and PFS (Table S3). Mutations in *B2M* (mPFS: 4.17 vs 10.78 months; HR = 6.96, 95% CI: 1.33-36.34, *P* = .007), *ZNF703* (4.17 vs 10.78 months; HR = 6.37, 95% CI: 1.23-32.95, *P* = .011), *KEAP1* (4.17 vs 10.78 months; HR = 8.45, 95% CI: 2.14-33.39, *P* < .001), and *TP53* (mPFS: 4.45 vs 11.20 months; HR = 3.78, 95% CI: 1.23-11.59, *P* = .012) were significantly associated with shorter PFS (Figure 3A-D). In contrast, *EP300* and *PIK3CA* mutations were associated with prolonged PFS (Figure 3E and F). Because no PFS events were observed among patients harboring *EP300* mutations, Firth’s penalized Cox regression was applied to reduce the statistical bias caused by sparse events and complete separation. After Firth correction, *EP300* mutation remained associated with favorable PFS after correction (mPFS: not reached vs 9.53 months, HR = 0.08, 95% CI: 6.59 × 10^−4^–0.61, *P* = .007). *PIK3CA* mutations were also significantly associated with prolonged PFS (mPFS: not reached vs 5.95 months, HR = 0.19, 95% CI: 0.07-0.49, *P* < .001).

**Figure 4:**
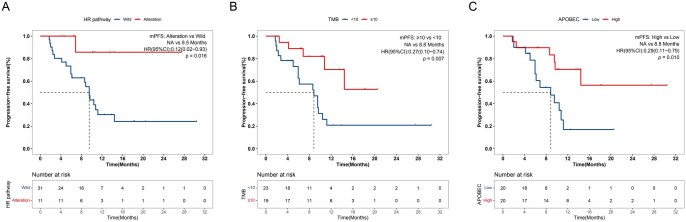
Progression-free survival according to homologous recombination pathway alteration, tumor mutational burden, and APOBEC signature. Kaplan-Meier curves comparing progression-free survival according to (A) homologous recombination pathway alteration status, (B) tumor mutational burden status, and (C) APOBEC signature level. For tumor mutational burden, patients were stratified using the prespecified cutoff of 10 mutations per megabase. HR pathway, homologous recombination pathway; TMB, tumor mutational burden; APOBEC, apolipoprotein B messenger RNA editing enzyme catalytic polypeptide-like; PFS, progression-free survival; mPFS, median progression-free survival; HR, hazard ratio; CI, confidence interval; NA, not available.

At the pathway level, HR pathway alterations were associated with favorable outcomes (mPFS: NR vs 9.53 months, HR = 0.12, 95% CI: 0.02-0.93, *P* = .016; Figure 4A). High TMB and high APOBEC signature level were also associated with prolonged PFS (TMB: HR = 0.27, 95% CI: 0.10-0.74, *P* = .007; APOBEC: HR = 0.29, 95% CI: 0.11-0.79, *P* = .010; Figure 4B and C).

Considering the reported relevance of *STK11* and the cGAS/STING pathway mutations to immunotherapy response in other tumor types, particularly non-small cell lung cancer,^34^^,^^35^ we further evaluated their alterations and potential association with PFS in our cohort. *STK11* mutations were detected in two patients (4.8%) and were not associated with PFS (HR = 2.20, 95% CI: 0.49-9.70, *P* = .290). No mutations were detected in cGAS/STING pathway-related genes (*IFNA6* and *IFNB1*) covered by the sequencing panel.

### Construction and evaluation of a genomic-based prognostic model

**Figure 5:**
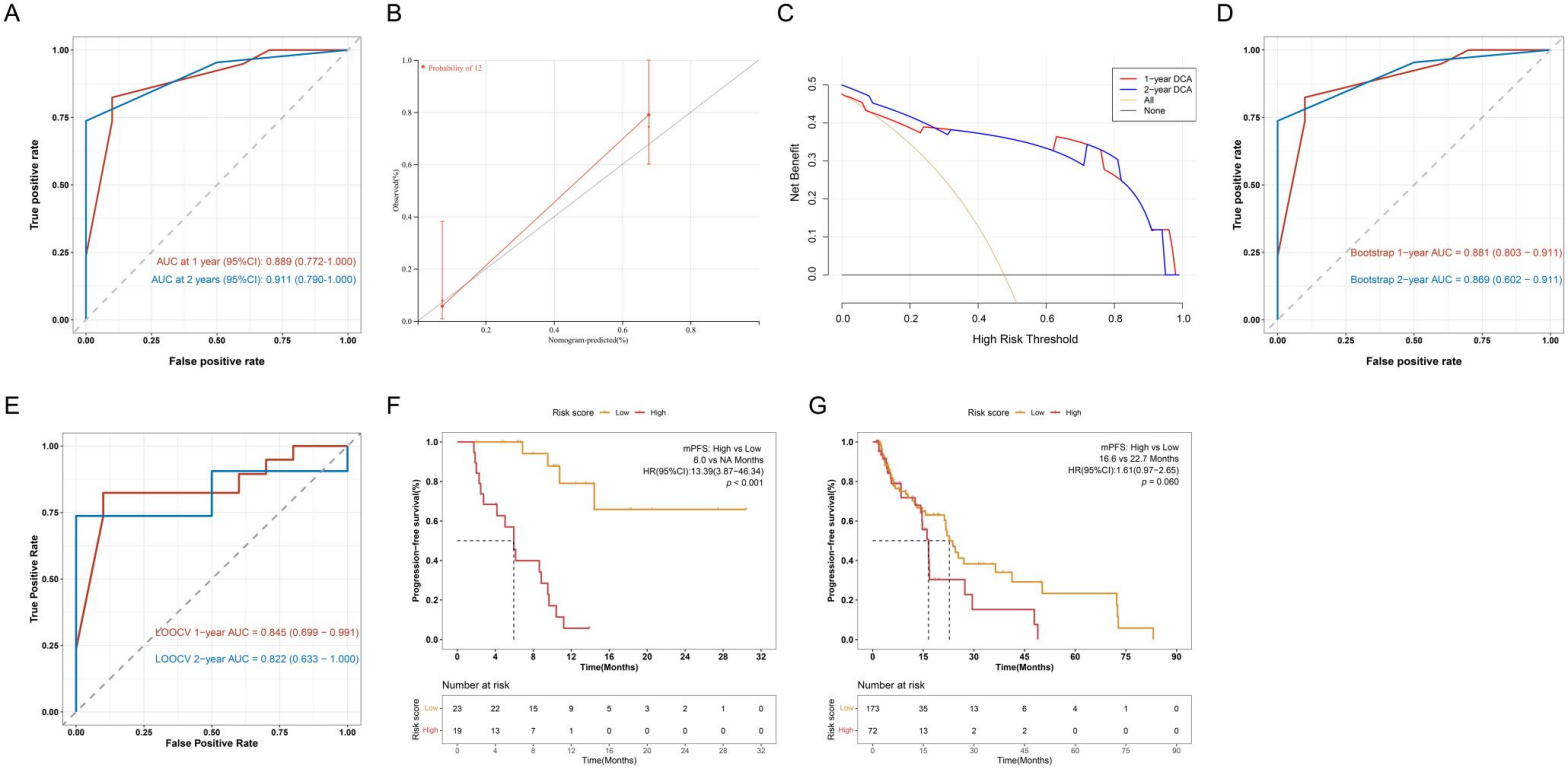
Construction, evaluation, and exploratory external assessment of the genomic risk model. (A) Time-dependent receiver operating characteristic curves for predicting 1-year and 2-year progression-free survival. (B) Calibration plot comparing predicted and observed progression-free survival probabilities. (C) Decision curve analysis evaluating the clinical net benefit of the model across different threshold probabilities. (D) Bootstrap-corrected time-dependent receiver operating characteristic curves. (E) Leave-one-out cross-validation-corrected time-dependent receiver operating characteristic curves. (F) Kaplan-Meier curve comparing progression-free survival between high- and low-risk groups in the study cohort. (G) Kaplan-Meier curve comparing progression-free survival between high- and low-risk groups in an exploratory pan-cancer immune checkpoint inhibitor-treated cohort. ROC, receiver operating characteristic; AUC, area under the curve; PFS, progression-free survival; DCA, decision curve analysis; LOOCV, leave-one-out cross-validation; ICI, immune checkpoint inhibitor; mPFS, median progression-free survival; HR, hazard ratio; CI, confidence interval; NA, not available.

LASSO regression identified five variables for model construction: *KEAP1*, *TP53*, *EP300*, *PIK3CA* mutations, and HR pathway alterations (Figure S2A). These features were incorporated into a multivariable Cox model to generate a prognostic risk score. The model demonstrated good discriminative ability, with a C-index of 0.810 (Figure S2B) and 1-year and 2-year AUCs of 0.889 and 0.911, respectively (Figure 5A). Calibration analysis showed generally consistent predicted and observed PFS probabilities, although interpretation was limited by sample size (Figure 5B). Decision curve suggested a higher net benefit than the treat-all and treat-none strategies across a broad range of threshold probabilities (Figure 5C). Internal validation yielded a bootstrap-corrected C-index was 0.790, with corrected AUCs of 0.881 and 0.869 for 1-year and 2-year PFS, respectively (Figure 5D). LOOCV yielded a corrected C-index of 0.778, with AUCs of 0.845 and 0.822 for 1-year and 2-year PFS, respectively (Figure 5E). These findings supported the internal consistency of the prognostic model.

Using the median risk score (−1.2652) as the cutoff value, patients were stratified into high- and low-risk groups (Figure S2C). High-risk patients exhibited significantly shorter PFS than low-risk patients (mPFS: 5.95 months vs not reached, HR = 13.39, 95% CI: 3.87-46.34, *P* < .001) (Figure 5F). As an exploratory external assessment, we applied the model to an independent pan-cancer cohort consisting of 249 ICI-treated patients from a public database,^19^ of whom 245 had available PFS data. As summarized in Table S4, the cohort included seven cancer types, predominantly melanoma (60.0%), non-small cell lung cancer (22.9%), and bladder cancer (11.0%), with smaller proportions of head and neck cancer (4.9%), anal cancer, small cell lung cancer, and soft tissue sarcoma (each 0.41%). Applying the same cutoff value, patients in the high-risk group had numerically shorter PFS than those in the low-risk group (mPFS: 16.55 vs 22.70 months, HR = 1.61, 95% CI: 0.97-2.65, *P* = .060; Figure 5G).

### Exploratory transcriptomic and tumor microenvironment analysis in the TCGA cohort

**Figure 6:**
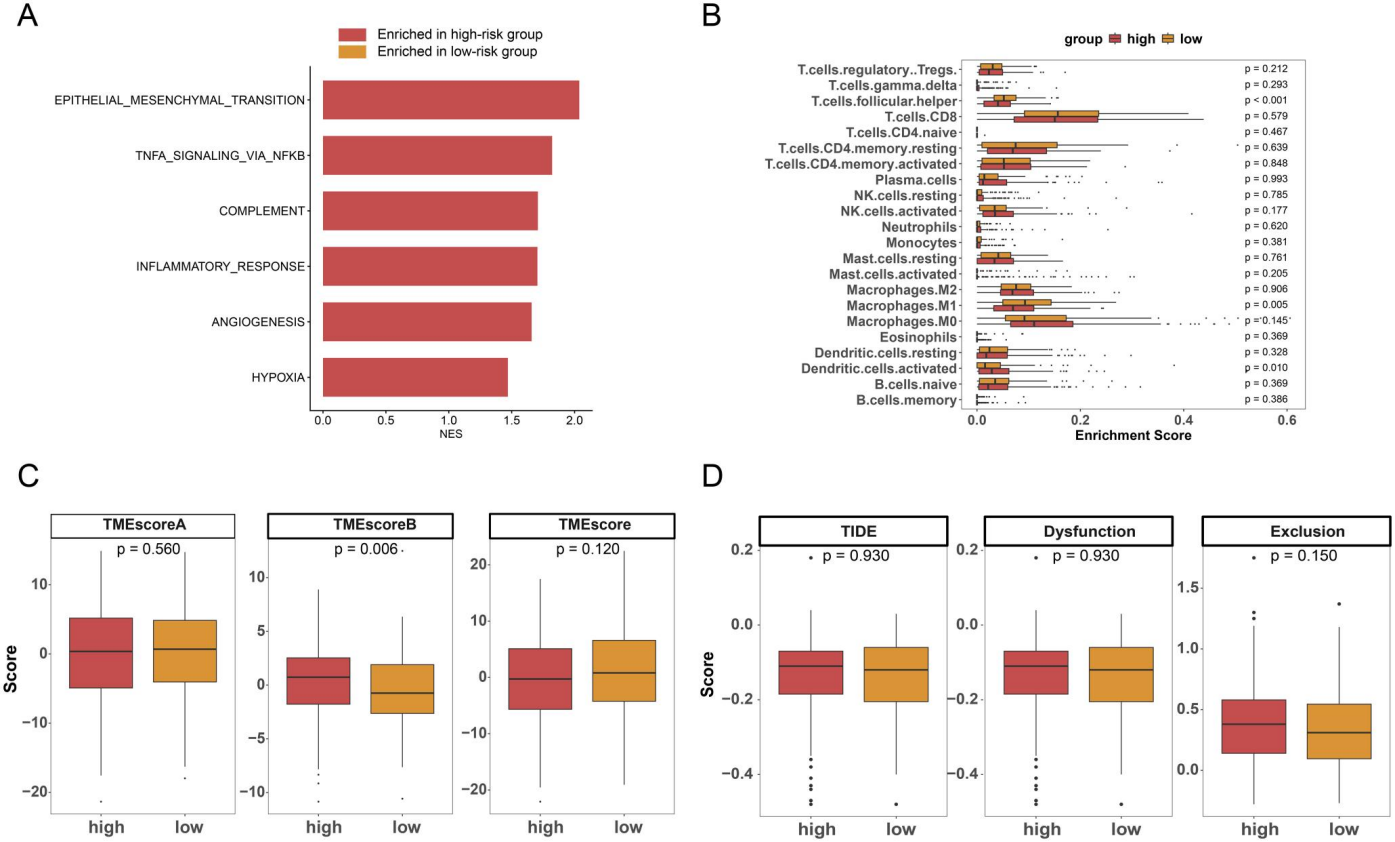
Exploratory transcriptomic and tumor immune microenvironment analyses using The Cancer Genome Atlas cervical cancer cohort. (A) Gene set enrichment analysis comparing high- and low-risk tumors. Red bars indicate pathways enriched in high-risk tumors, and orange bars indicate pathways enriched in low-risk tumors. (B) Immune cell infiltration profiles computationally inferred between high- and low-risk tumors. (C) Tumor microenvironment scores according to risk group. (D) Tumor Immune Dysfunction and Exclusion scores according to risk group. TCGA, The Cancer Genome Atlas; GSEA, gene set enrichment analysis; NES, normalized enrichment score; TME, tumor microenvironment; TIDE, Tumor Immune Dysfunction and Exclusion; NK cells, natural killer cells; CD8, cluster of differentiation 8; TNFα, tumor necrosis factor-alpha; NF-κB, nuclear factor-kappa B.

The prognostic model was applied to 286 TCGA cervical cancer samples with matched DNA and RNA data. Computational transcriptomic analyses high-risk tumors were enriched in angiogenesis, epithelial-mesenchymal transition (EMT), hypoxia, inflammatory response, and TNFα/NF-κB signaling pathways (all FDR-adjusted *P* < 0.050, Figure 6A). CIBERSORT analysis showed lower inferred level of M1 macrophages and follicular helper T cells levels but higher activated dendritic cells levels in the high-risk group (*P* = .005, *P* < .001, and *P* = .010, respectively; Figure 6B). TMEscoreB was higher in the high-risk group (*P* = .006; Figure 6C), whereas TIDE, dysfunction, and exclusion scores did not differ significantly (Figure 6D).

We further investigated pathway activity, immune cell infiltration, and TME-related scores according to the five model-related genomic factors. GSEA showed distinct pathway patterns across these alterations (FDR-adjusted *P* < .050; Figure S3A-E). *PIK3CA*-mutant tumors showed downregulation of multiple oncogenic and immune-related pathways; *EP300* mutations were associated with decreased EMT activity; HR pathway alterations were linked to upregulation of interferon-α and interferon-γ response pathways and downregulation EMT; *TP53* mutations showed broad suppression of immune-related signaling; and *KEAP1*-mutant tumors showed enrichment of reactive oxygen species and DNA repair pathways.

Immune infiltration analysis showed alteration-specific patterns across the five genomic factors (Figure S4A-E). *PIK3CA*-mutant tumors showed higher follicular helper T-cell infiltration (*P* = .001). *EP300*-mutant tumors showed higher M1 macrophage infiltration (*P* = .001) but lower activated dendritic cell infiltration (*P* = .003). HR pathway-altered tumors showed higher follicular helper T-cell and CD8^+^ T-cell infiltration (*P* = .005 and *P* = .037, respectively) but lower neutrophil infiltration (*P* = .011). *TP53*-mutant tumors showed lower γδ T-cell and activated dendritic cell infiltration (*P* = .022 and *P* = .036, respectively), whereas *KEAP1*-mutant tumors showed lower activated natural killer cell infiltration (*P* = .010).

TME-related analyses also revealed alteration-specific patterns (Figure S5-E). *PIK3CA*-mutant tumors showed lower TMEscoreB (*P* = .022). *EP300*-mutant tumors showed lower TMEscoreB (*P* = .003) and higher overall TME scores (*P* = .018), whereas HR pathway-altered tumors showed higher overall TME scores (*P* = .040). *TP53*-mutant tumors showed lower TMEscoreB (*P* = .012), as well as lower TIDE and dysfunction scores (both *P* = .026) but higher exclusion scores (*P* = .04). *KEAP1*-mutant tumors showed no significant changes in TME or exhaustion-related scores.

## Discussion

In this study, we conducted an integrative genomic analysis of patients with recurrent or metastatic cervical cancer receiving ICIs to identify molecular correlates of therapeutic response and develop a prognostic model for PFS. The median PFS of 10.4 months in our cohort was comparable to that reported in KEYNOTE-826, in which pembrolizumab plus chemotherapy with or without bevacizumab improved outcomes in patients with PD-L1 CPS ≥1.^7^

Frequent alterations were observed in *PIK3CA*, *EP300*, and *TERT*, with *PIK3CA* and *EP300* mutations associated with higher TMB and prolonged PFS, suggesting that these alterations may co-occur with mutagenic processes and increased tumor immunogenicity.^12^^,^^36^ In contrast, *B2M*, *KEAP1*, and *TP53* mutations were associated with shorter PFS, consistent with their reported roles in immune evasion, impaired antigen presentation, and resistance to immunotherapy in other tumor types.^37–40^ Pathway-level alterations in PI3K, NOTCH, and HR pathways were associated with favorable outcomes, supporting the relevance of pathway-based genomic features in ICI response.^41–43^

Exploratory analyses using TCGA transcriptomic data suggested that the genomic risk score and model-related alterations were associated with distinct tumor immune microenvironmental features. Gene-specific analyses suggest that *PIK3CA*, *EP300*, HR pathway, *KEAP1*, and *TP53* alterations may be associated with distinct immune microenvironmental features. However, these TCGA-based findings were derived from baseline bulk transcriptomic data and computational deconvolution, and should therefore be interpreted as hypothesis-generating rather than mechanistic validation.

We developed a genomic-based prognostic model incorporating *KEAP1*, *TP53*, *EP300*, *PIK3CA* mutations, and HR pathway alterations, which stratified patients into high- and low-risk groups. Major clinical variables, such as PD-L1 CPS, HPV status, histological subtype, and treatment regimen, were not significantly associated with PFS and were not incorporated into the final model to avoid increasing model instability under limited sample-size conditions. Nevertheless, the ICI response is multifactorial, and larger cohorts are needed to determine whether clinical variables provide additional predictive value beyond genomic features. External validation in an independent pan-cancer ICI-treated cohort showed a similar directional trend, although the result did not reach statistical significance. Therefore, this finding should be interpreted strictly as exploratory, and further validation in larger cervical cancer-specific cohorts is strongly warranted before any broader applicability can be considered. Applying our genomic risk score to TCGA cervical cancers, computational transcriptomic and immune deconvolution analyses suggested that high-risk tumors were associated with angiogenesis, EMT, hypoxia, inflammatory response, TNFα/NF-κB signaling, and altered immune cell infiltration patterns at baseline. These features are consistent with previously reported mechanisms of ICI resistance, including TGF-β/EMT-driven T-cell exclusion and angiogenesis-related immunosuppression,^44^^,^^45^ thereby providing a biological context for the poorer outcomes observed in the high-risk group. However, these findings were derived from baseline TCGA tumor transcriptomic data and computational immune deconvolution analyses, rather than paired longitudinal samples or experimental immune profiling. Because post-ICI treatment samples were not available in the present study, treatment-induced molecular or immune microenvironmental changes could not be evaluated. Future studies incorporating paired pre- and post-treatment specimens are warranted. These TCGA-derived findings should be interpreted as hypothesis-generating rather than direct mechanistic validation.

Several limitations should be acknowledged. First, the small sample size limited statistical power and increased the risk of overfitting. Although LASSO regression, bootstrap resampling, and LOOCV were applied, these approaches cannot fully eliminate model instability. Second, our prognostic model was developed based on PFS rather than OS, as OS data were immature at the time of analysis. While PFS may reflect early treatment benefit and disease control, its correlation with long-term survival in this specific cohort remains to be confirmed. Third, the retrospective nature of this study precludes causal inferences between genomic alterations and clinical outcomes and limits the availability of comprehensive treatment-related toxicity data, including immune-related adverse events. Consequently, the potential correlation between genomic alterations and immunotherapy-induced toxicity could not be evaluated. Finally, post-ICI tumor samples were unavailable, preventing assessment of treatment-induced molecular or immune microenvironmental changes. Therefore, the model should be considered exploratory, and larger prospective studies with independent cervical cancer cohorts, extended follow-up, mature OS data, and paired pre- and post-treatment specimens are needed to validate its stability, clinical applicability, and relationship with long-term outcomes.

## Conclusion

In summary, our study identified genomic alterations associated with immunotherapy outcomes in recurrent or metastatic cervical cancer and developed an exploratory genomic risk model for PFS stratification. Integrative transcriptomic and immune microenvironment analyses provided potential biological context for the observed clinical associations. Given the limited sample size and exploratory nature of the model, further validation in larger, independent cervical cancer cohorts is warranted before clinical implementation.

## Supplementary Material

oyag236_Supplementary_Data

## Data Availability

The datasets generated and/or analyzed during this current study are not publicly available due to the privacy protection policy of personal medical data at our institution but are available from the corresponding author upon reasonable request.
