# Supplementary material for: Genomic determinants and an exploratory prognostic model for immunotherapy outcomes in recurrent or metastatic cervical cancer
Source: Oncologist. 2026 Jun 22;31(7):oyag236. doi: 10.1093/oncolo/oyag236 (PMC13331280; doi:10.1093/oncolo/oyag236)
Supplement: oyag236_Supplementary_Data [file oyag236_supplementary_data.zip › Table S4-2.docx]

**Supplemental Table S4. Characteristics of patients in the external validation cohort treated with immune checkpoint inhibitors.**

| **Characteristics** | **Total (N=245)** |
| --- | --- |
| **Sex** |  |
| Male | 151 61.6%) |
| Female | 94 (38.4) |
| **Cancer type** |  |
| Anal Cancer | 1(0.41%) |
| Bladder Cancer | 27(11.02%) |
| Head and Neck Cancer | 12(4.90%) |
| Melanoma | 147(60.00%) |
| Non-Small Cell Lung Cancer | 56(22.86%) |
| Small Cell Lung Cancer | 1(0.41%) |
| Soft Tissue Sarcoma | 1(0.41%) |
| **Treatment** |  |
| anti-CTLA-4 | 141(57.55%) |
| anti-CTLA-4 + anti-PD-1/PD-L1 | 10(4.08%) |
| anti-PD-1/anti-PD-L1 | 94(38.37%) |

Abbreviations: CTLA, cytotoxic T-Lymphocyte–associated protein; PD-L1, programmed death-ligand 1
