# Supplementary material for: Genomic determinants and an exploratory prognostic model for immunotherapy outcomes in recurrent or metastatic cervical cancer
Source: Oncologist. 2026 Jun 22;31(7):oyag236. doi: 10.1093/oncolo/oyag236 (PMC13331280; doi:10.1093/oncolo/oyag236)
Supplement: oyag236_Supplementary_Data [file oyag236_supplementary_data.zip › Table S2-2.docx]

**Supplemental Table S2.** **Univariable Cox regression analysis of clinical features for progression-free survival in patients with recurrent/metastatic cervical cancer.**

| **Characteristic** | **HR (95% CI)** | **P value** |
| --- | --- | --- |
| **Age** |  |  |
| >50 vs ≤50 | 1.05(0.44-2.50) | 0.914 |
| **HPV status** |  |  |
| Positive vs Negative | 1.06(0.35-3.22) | 0.918 |
| **Histological subtype** |  |  |
| CADC vs CSCC | 0.61(0.14-2.66) | 0.510 |
| **PD-L1 (CPS)** |  |  |
| ≥5 | 1.44(0.60-3.42) | 0.411 |
| ≥10 | 0.93(0.36-2.41) | 0.876 |
| ≥15 | 0.77(0.28-2.10) | 0.605 |
| ≥20 | 1.43(0.47-4.33) | 0.525 |
| ≥25 | 1.08(0.25-4.67) | 0.917 |
| ≥30 | 0.69(0.09-5.20) | 0.719 |
| ≥35 | 0.95(0.12-7.23) | 0.961 |
| **Treatment regimen** |  |  |
| Chemotherapy + Immunotherapy + Bevacizumab vs Chemotherapy + Immunotherapy | 1.81(0.77-4.30) | 0.170 |

Abbreviations: CADC: cervical adenocarcinoma; CSCC: cervical squamous cell carcinoma; HR: Hazard Ratio; CI: Confidence Interval, HPV, human papillomavirus; CPS, combined positive score; PD-L1, programmed death-ligand 1
