# Supplementary material for: Genomic determinants and an exploratory prognostic model for immunotherapy outcomes in recurrent or metastatic cervical cancer
Source: Oncologist. 2026 Jun 22;31(7):oyag236. doi: 10.1093/oncolo/oyag236 (PMC13331280; doi:10.1093/oncolo/oyag236)
Supplement: oyag236_Supplementary_Data [file oyag236_supplementary_data.zip › Table S3-2.docx]

**Supplemental Table S3**. **Univariable Cox regression analysis of gene mutations, signaling pathway alterations, and mutational signatures associated with progression-free survival in patients with recurrent or metastatic cervical cancer receiving immunotherapy.**

| **Characteristic** | **HR (95% CI)** | **P value** |
| --- | --- | --- |
| ***PIK3CA*** |  |  |
| Alteration vs wild type | 0.19(0.07-0.49) | 0.001 |
| ***KEAP1*** |  |  |
| Alteration vs wild type | 8.45(2.14-33.39) | <0.001 |
| ***B2M*** |  |  |
| Alteration vs wild type | 6.96(1.33-36.34) | 0.007 |
| ***ZNF703*** |  |  |
| Alteration vs wild type | 6.37(1.23-32.95) | 0.011 |
| ***TP53*** |  |  |
| Alteration vs wild type | 3.78(1.23-11.59) | 0.012 |
| ***EP300*** |  |  |
| Alteration vs wild type | 0.08 (6.59e⁻⁴-0.61) | 0.007 |
| **HR pathway** |  |  |
| Alteration vs wild type | 0.12(0.02-0.93) | 0.016 |
| **PI3K pathway** |  |  |
| Alteration vs wild type | 0.38(0.16-0.91) | 0.025 |
| **NOTCH pathway** |  |  |
| Alteration vs wild type | 0.36(0.14-0.92) | 0.026 |
| **TMB** |  |  |
| ≥10 vs <10 | 0.27(0.10-0.74) | 0.007 |
| **CIS** |  |  |
| High vs Low | 2.06(0.86-4.95) | 0.099 |
| **APOBEC signature ratio** |  |  |
| High vs Low | 0.29(0.11-0.79) | 0.01 |
| **Age signature ratio** |  |  |
| High vs Low | 1.82(0.74-4.51) | 0.187 |
| **MMR deficiency signature ratio** |  |  |
| High vs Low | 0.57(0.20-1.60) | 0.279 |
| **Ultraviolet signature ratio** |  |  |
| High vs Low | 0.59(0.21-1.63) | 0.303 |
| **Temozolomide signature ratio** |  |  |
| High vs Low | 0.42(0.06-3.20) | 0.392 |
| **BRCA signature ratio** |  |  |
| High vs Low | 0.48(0.06-3.61) | 0.466 |
| **Smoking signature ratio** |  |  |
| High vs Low | 0.56(0.07-4.27) | 0.574 |
| **POLE signature ratio** |  |  |
| High vs Low | 1.05(0.40-2.77) | 0.92 |

Abbreviation: HR, hazard ratio; CI, confidence interval; TMB, tumor mutational burden; CIS, chromosomal instability score
