# Supplementary figures and images for: Genomic determinants and an exploratory prognostic model for immunotherapy outcomes in recurrent or metastatic cervical cancer
Source: Oncologist. 2026 Jun 22;31(7):oyag236. doi: 10.1093/oncolo/oyag236 (PMC13331280; doi:10.1093/oncolo/oyag236)

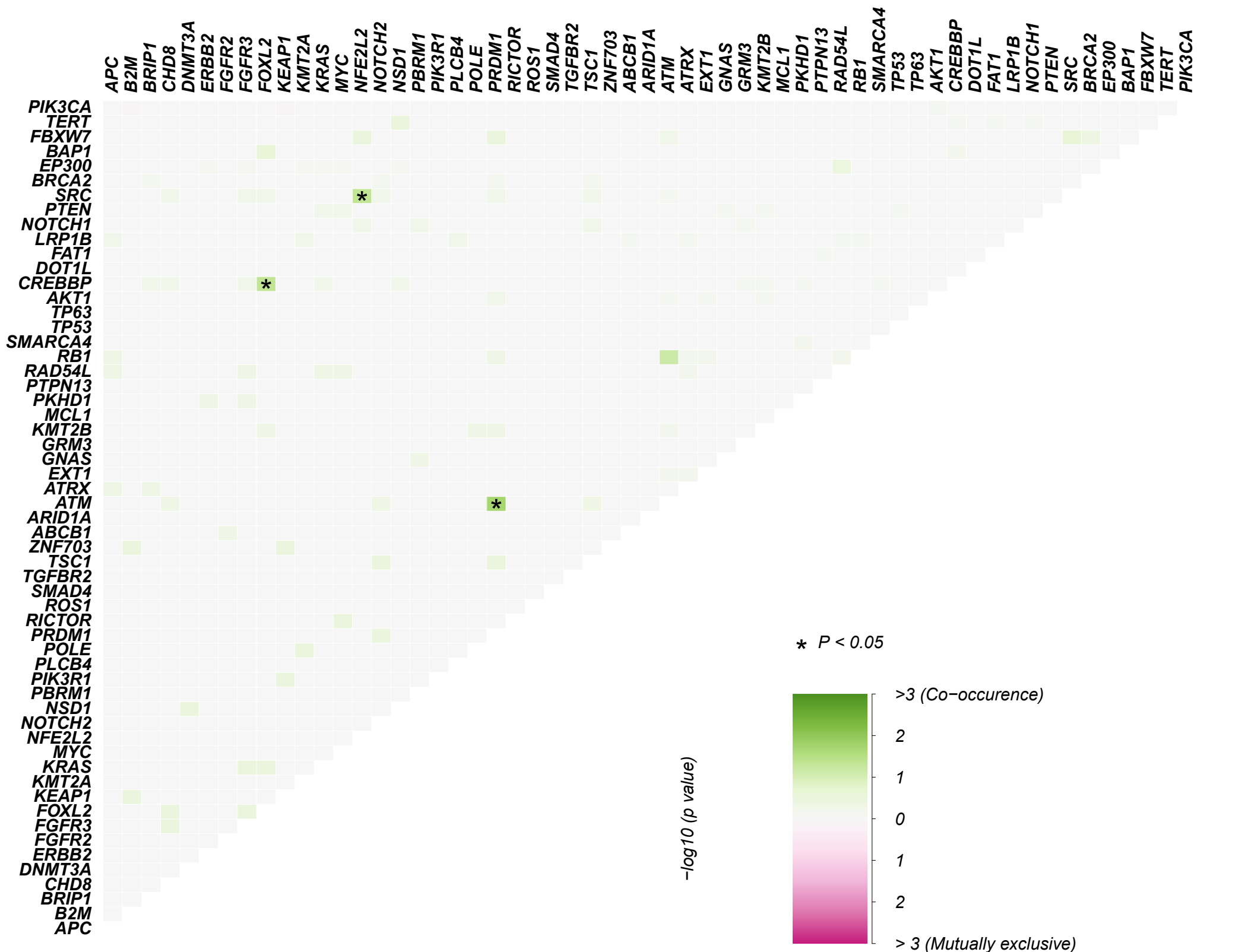

Supplement: oyag236_Supplementary_Data [file oyag236_supplementary_data.zip › Figure S1R.pdf]

A

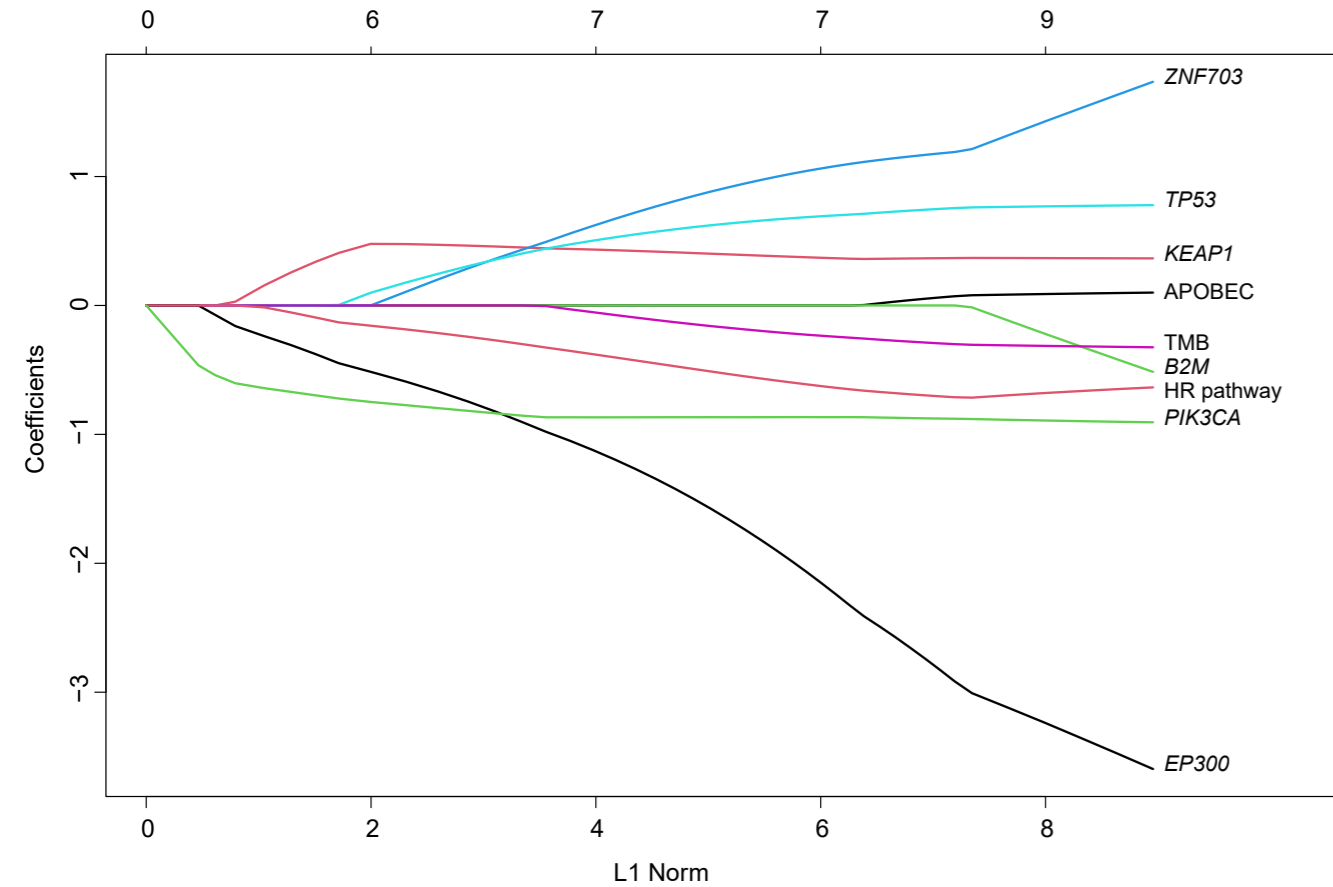

B

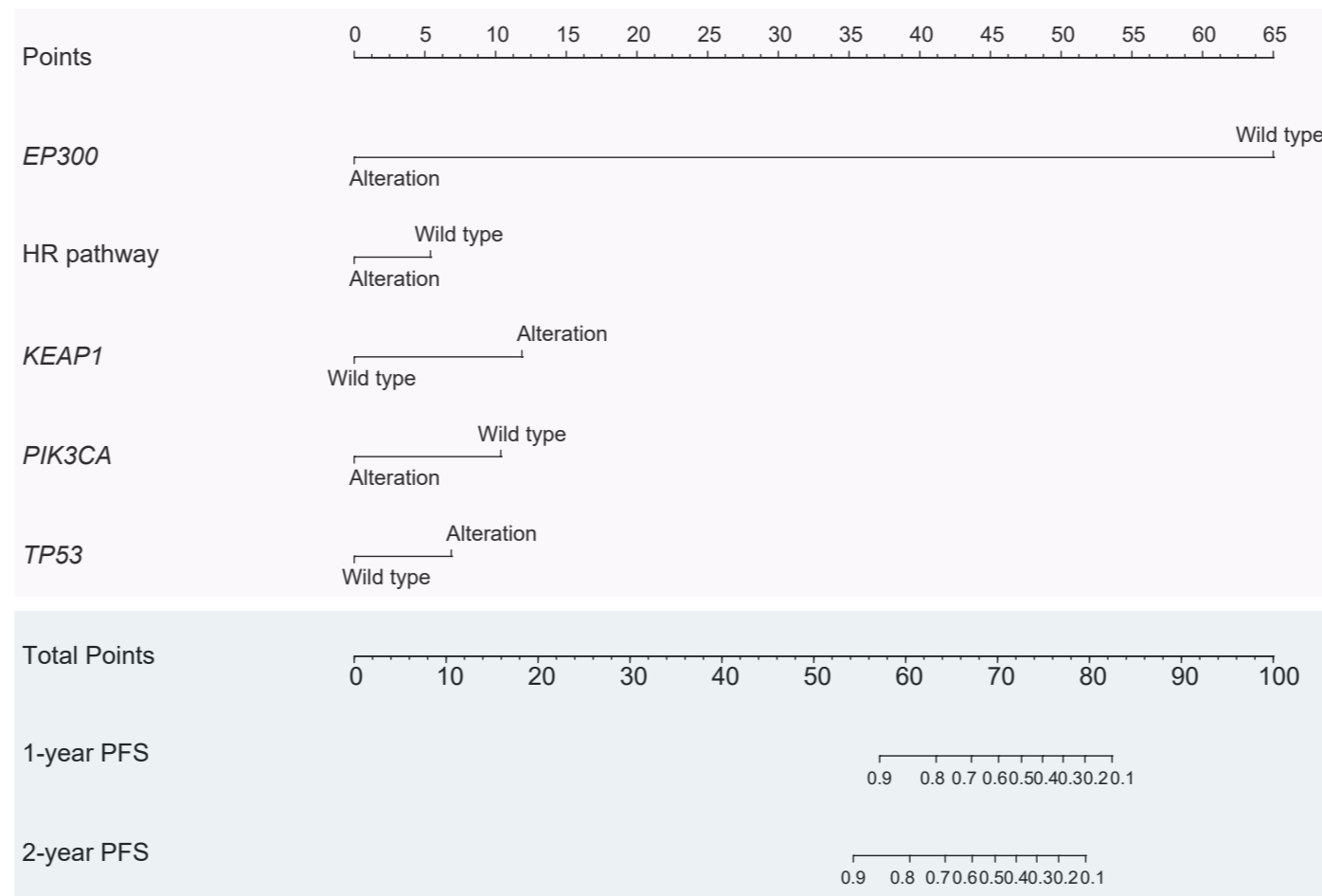

C

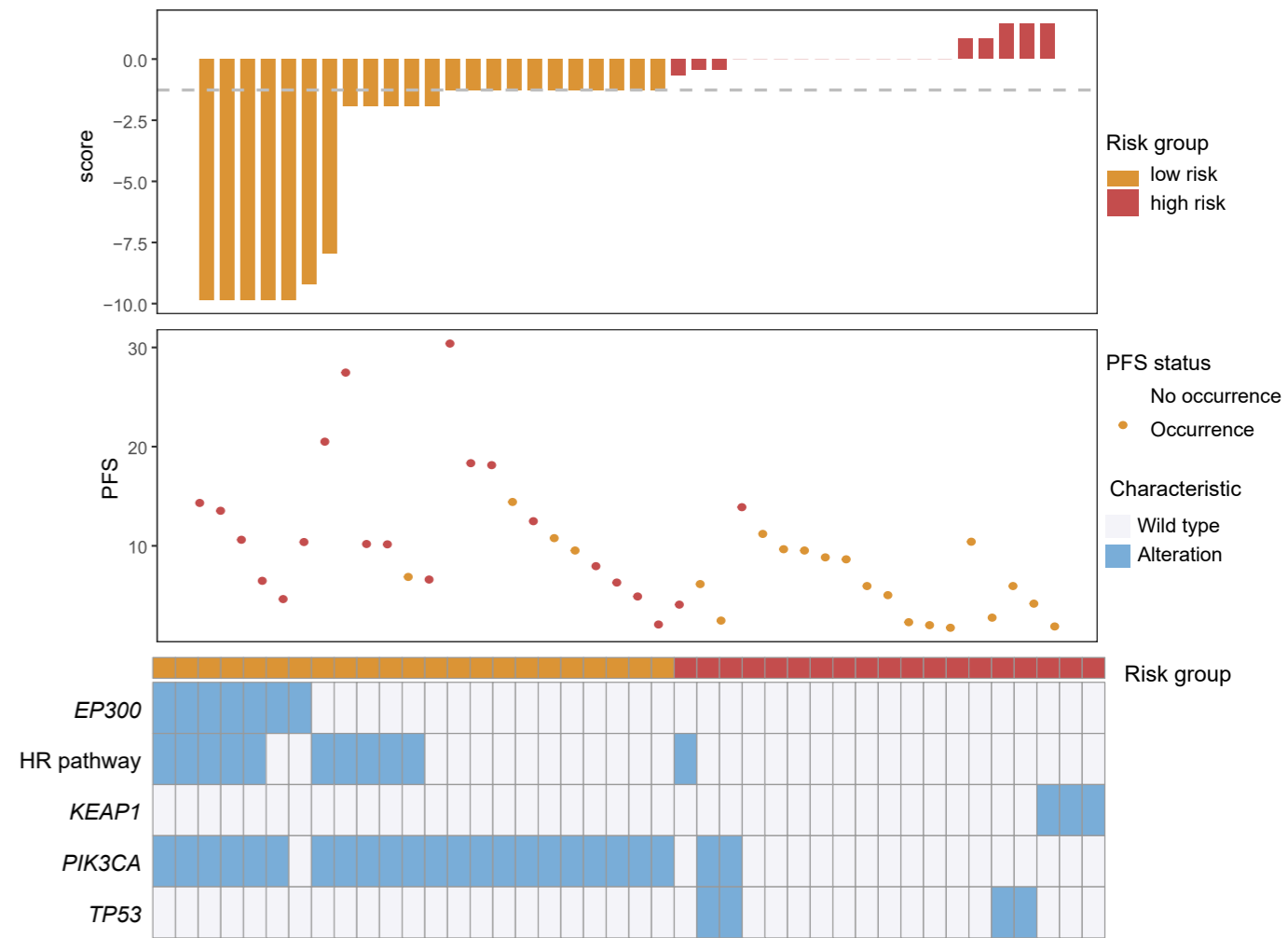

Supplement: oyag236_Supplementary_Data [file oyag236_supplementary_data.zip › Figure S2-0525.pdf]

A

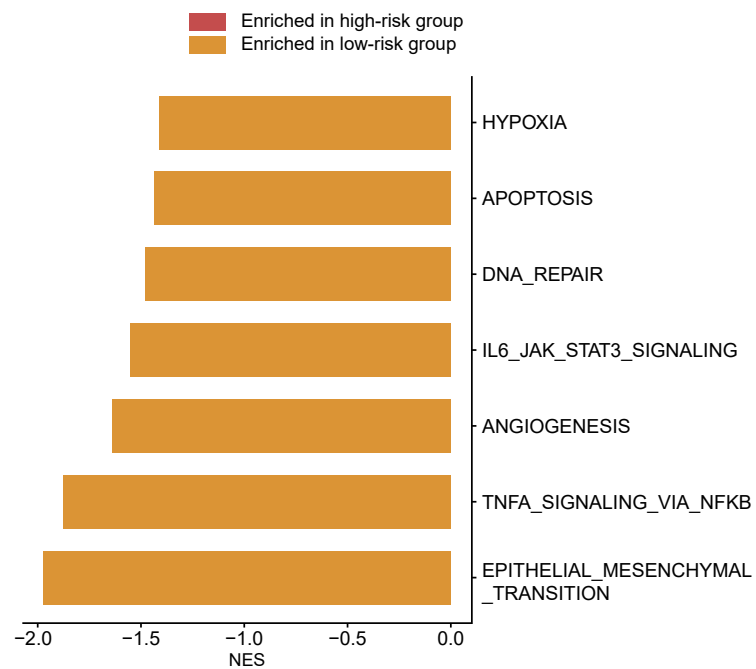

B

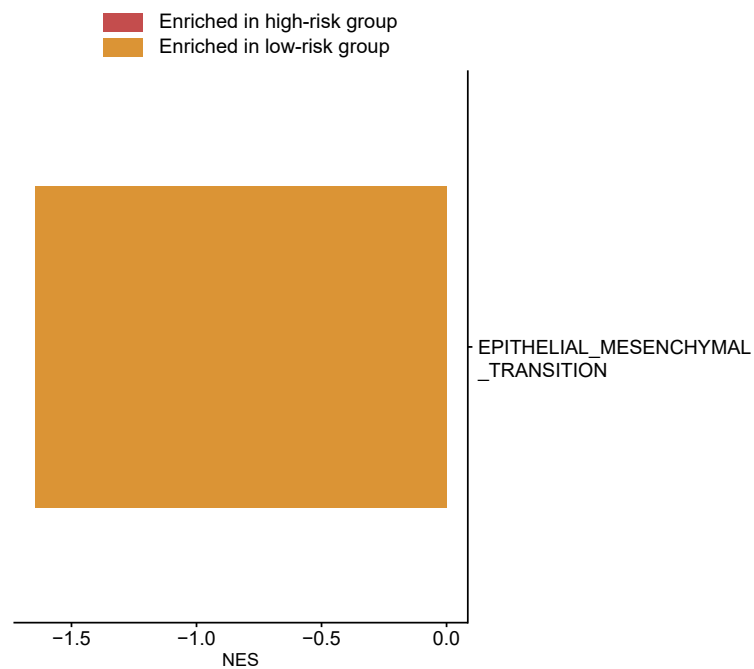

C

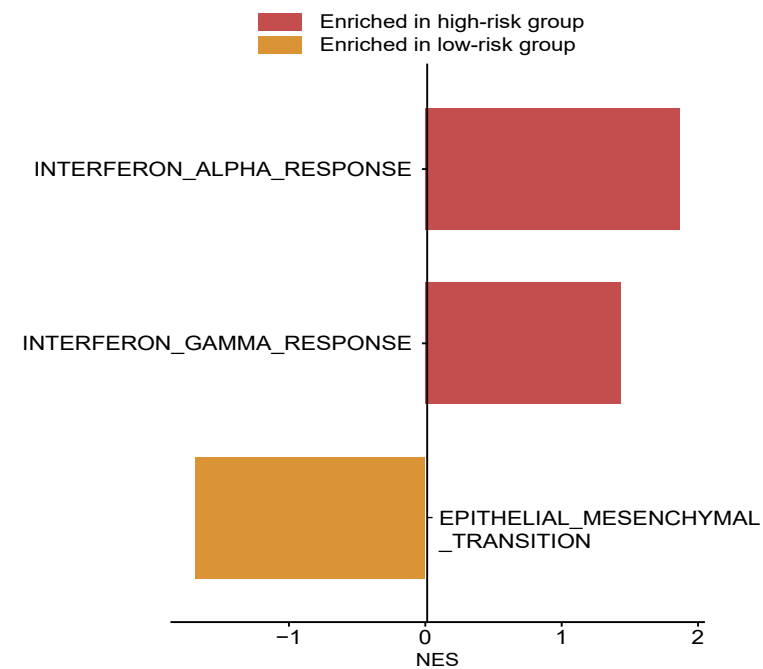

D

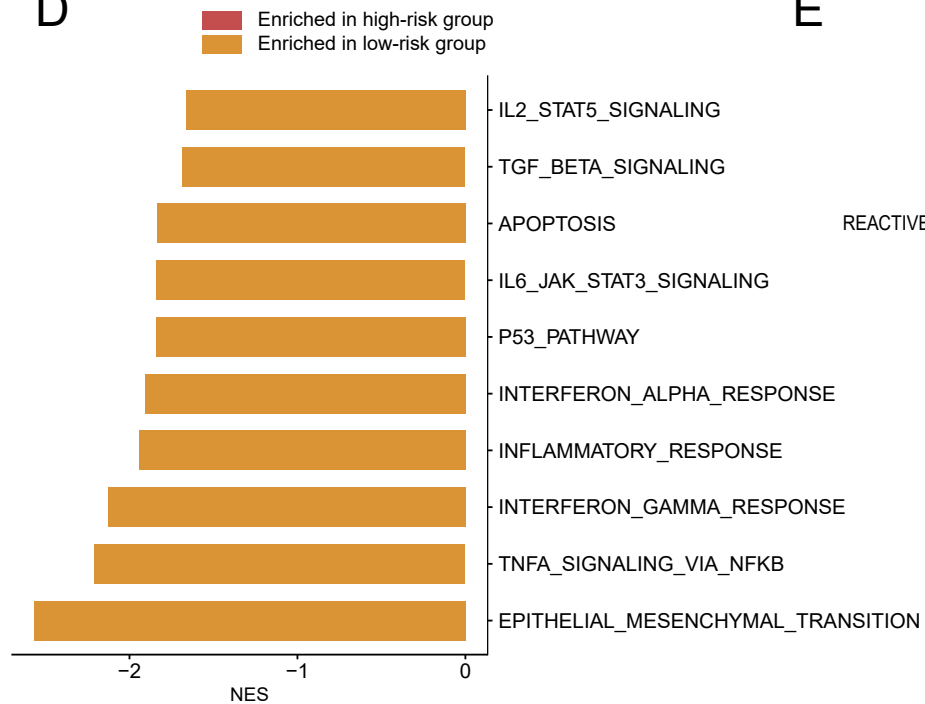

E

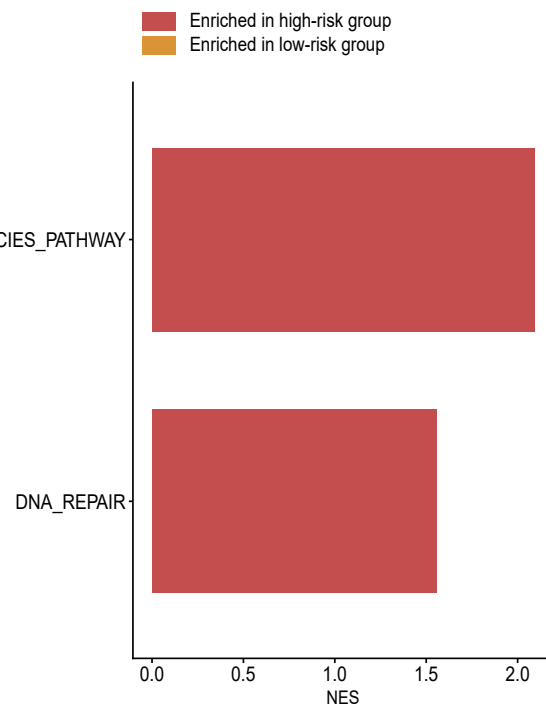

Supplement: oyag236_Supplementary_Data [file oyag236_supplementary_data.zip › Figure S3 v2-0525.pdf]

A

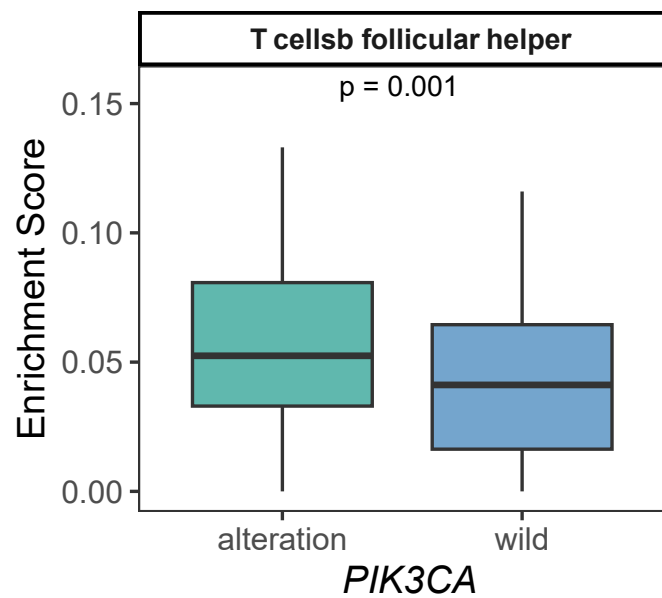

B

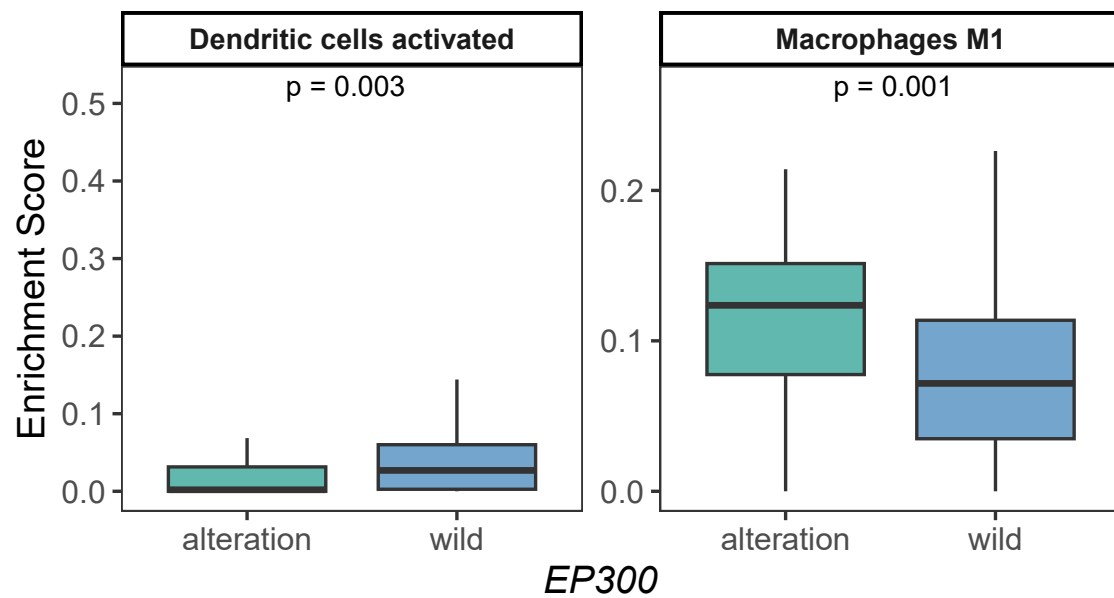

C

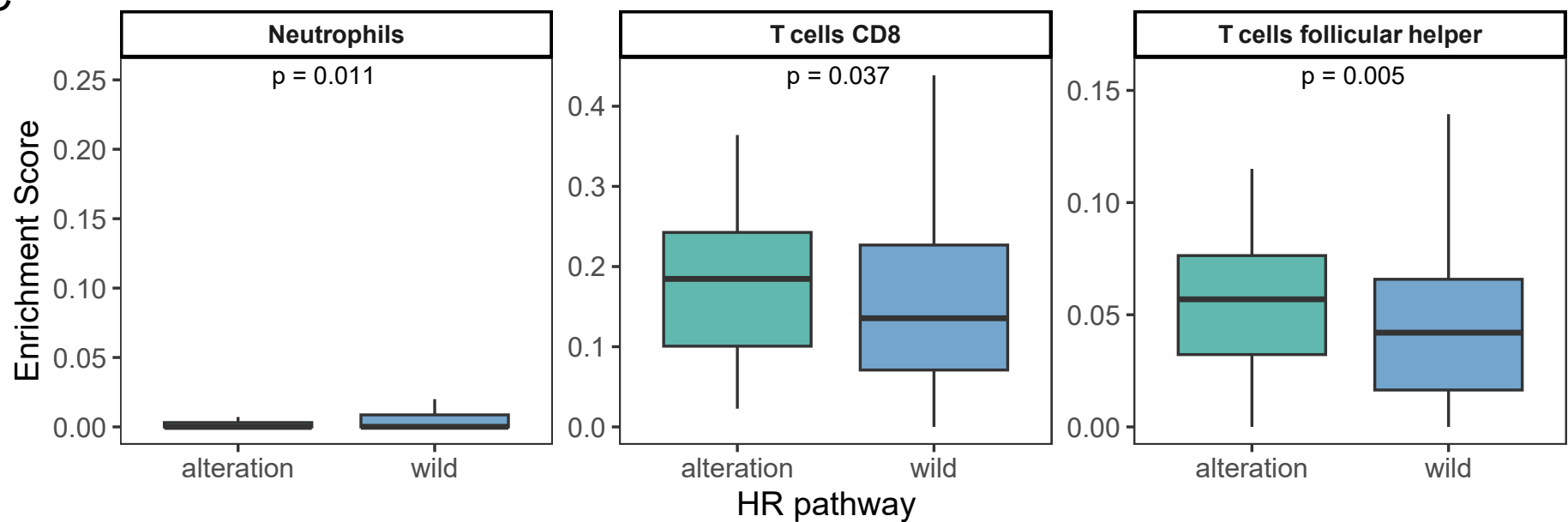

D

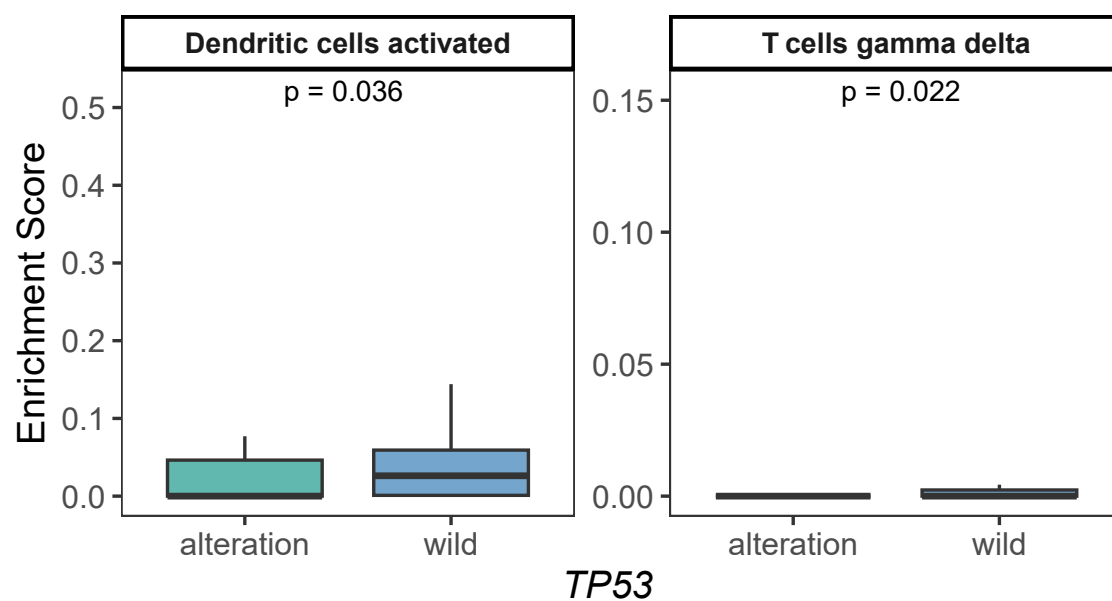

E

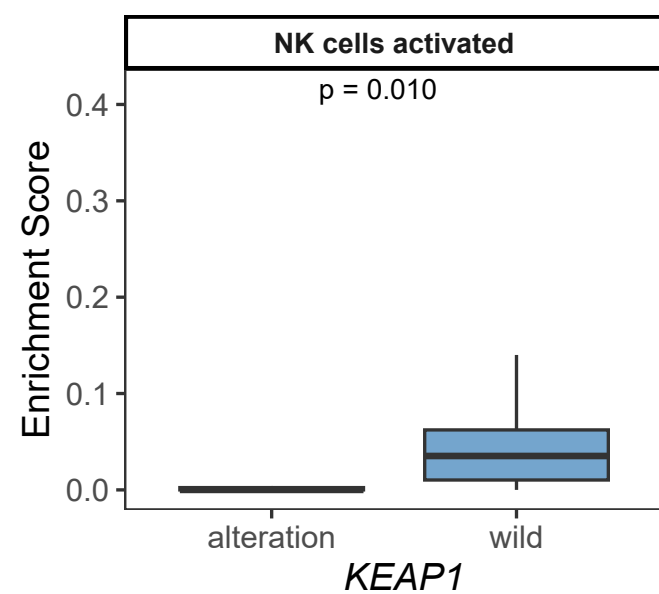

Supplement: oyag236_Supplementary_Data [file oyag236_supplementary_data.zip › Figure S4 v2-0525.pdf]

A

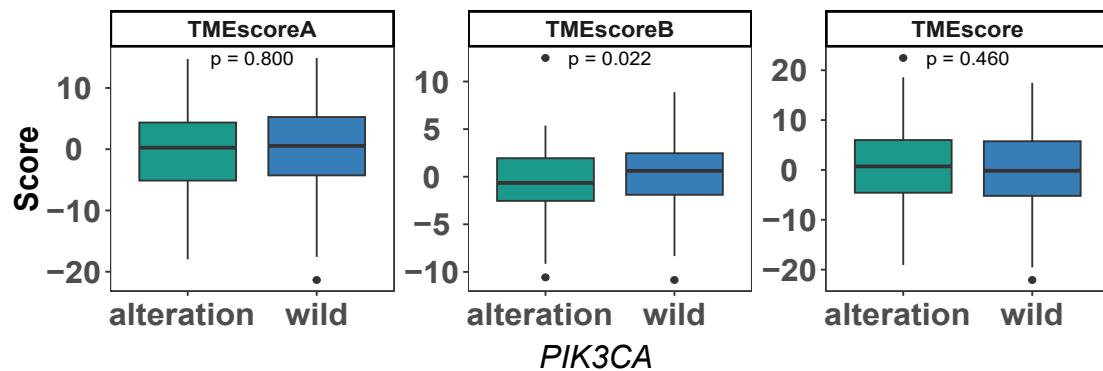

B

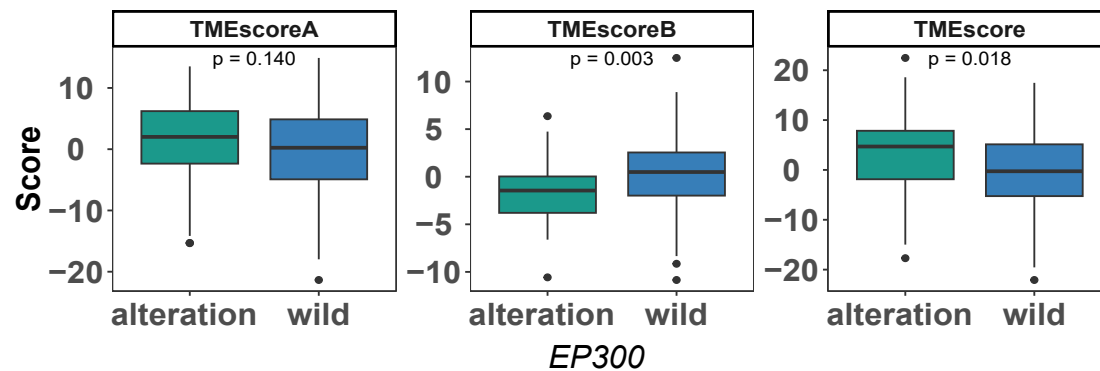

C

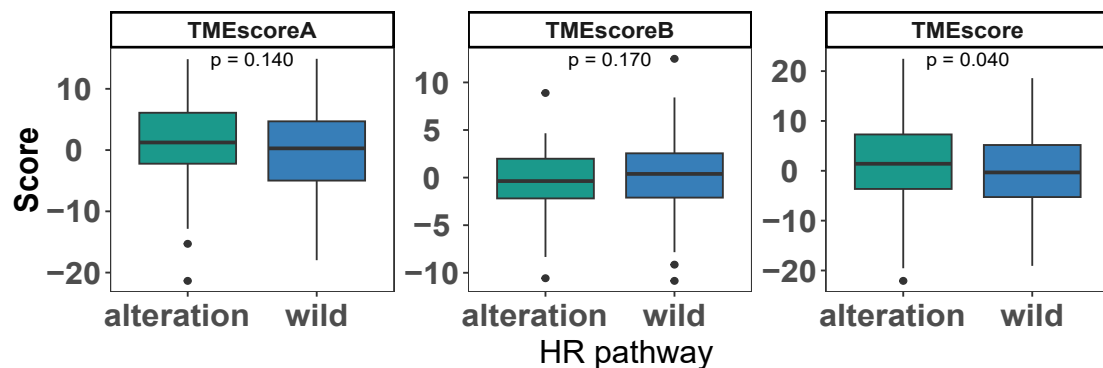

D

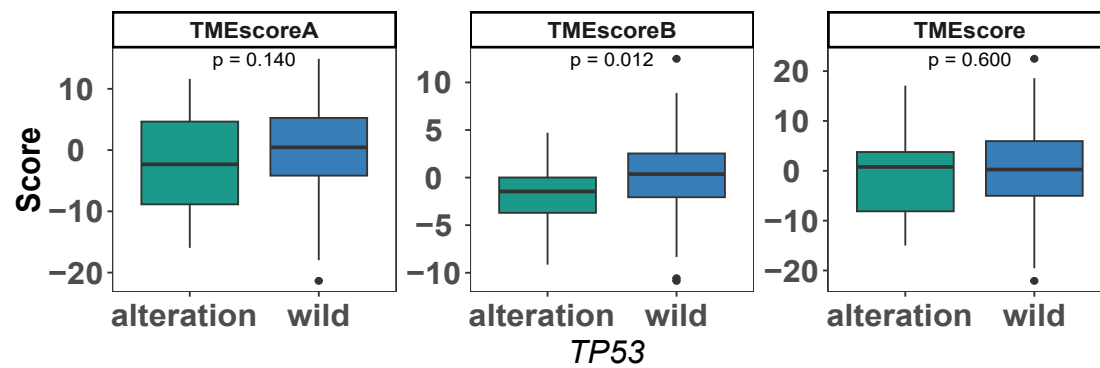

E

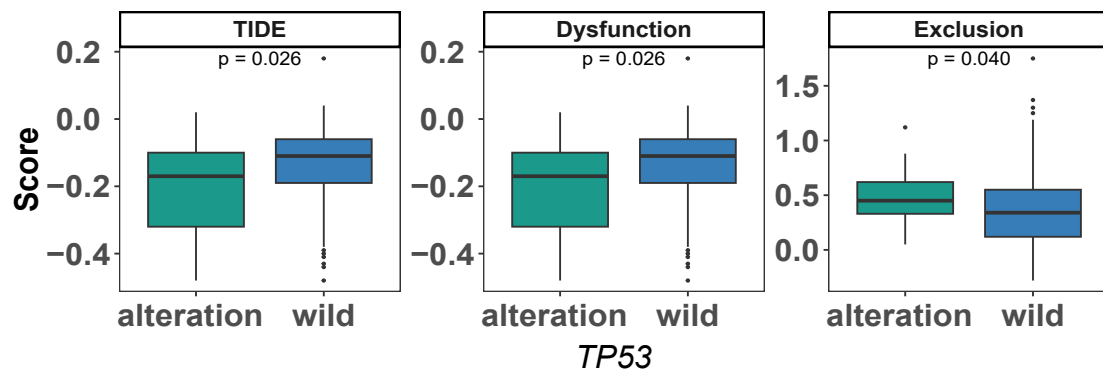

Supplement: oyag236_Supplementary_Data [file oyag236_supplementary_data.zip › Figure S5 v2-0525.pdf]
